# Micromegaly: a distinct clinical entity? Insights from a monocentric cohort study

**DOI:** 10.3389/fendo.2026.1874123

**Published:** 2026-07-20

**Authors:** Alessandra Mangone, Giulia Carosi, Elisa Sala, Giusy Marra, Giulia Del Sindaco, Roberta Mungari, Arianna Cremaschi, Veronica Lotito, Erika Peverelli, Emanuele Ferrante, Giovanna Mantovani

**Affiliations:** 1 Department of Clinical Sciences and Community Health, Dipartimento di Eccellenza 2023-2027, University of Milan, Milan, Italy; 2 Endocrinology Unit, Fondazione IRCCS Ca’ Granda Ospedale Maggiore Policlinico, Milan, Italy

**Keywords:** acromegaly, GH nadir, growth hormone, insulin growth factor 1, micromegaly

## Abstract

**Background:**

The term “micromegaly” historically refers to patients with acromegalic features, elevated IGF-1, but normal growth hormone (GH) levels. The physiological mechanism, the appropriate clinical and treatment approaches, as well as its existence as a separate clinical entity are not well-defined.

**Methods:**

We retrospectively collected clinical and hormonal data from 30 patients with acromegaly and 30 with micromegaly (displaying high IGF-1 but GH < 0.4 µg/L after glucose load), matched for age and sex. Data about acromegaly related comorbidities were collected (goiter, colonic polyps, malignancies, hypertension, cardiopathy, obstructive-sleep-apnea, carpal tunnel, and hyperglycemia). We performed a laboratory analysis of pituitary tissue samples from both groups, examining GH isoform expression and proliferative rate. We compared data of the two groups and described follow-up and treatment response in micromegalic patients.

**Results:**

Patients with acromegaly exhibited higher IGF-1 values at diagnosis (+8.3 vs. +3.2 SDS, *p* < 0.01) and higher GH nadir levels after glucose load (6 vs. 0.15 μg/L, *p* < 0.01). All patients with acromegaly had a detectable pituitary adenoma (70% macroadenomas), whereas 52% of patients with micromegaly showed no evidence of pituitary adenoma. Despite different GH/IGF-1 values, the prevalence of most comorbidities was similar in both groups, except for valve disease and diabetes mellitus, which were more frequent in acromegaly (valvopathy: 42.9% vs. 16.7%, *p* = 0.006; diabetes: 56.7% vs. 23.3%, *p* = 0.016, respectively). Laboratory analyses in patients with micromegaly indicated a lower proliferative profile in tumor cells (D3 cyclin expression).

**Conclusion:**

Patients with a clinical diagnosis of acromegaly and high IGF-1 levels but a GH nadir < 0.4 µg/L following glucose load exhibit a high burden of comorbidities and therefore require appropriate screening and follow-up. Therapeutic strategies in these cases should be individualized, considering the frequent absence of neuroradiological findings, which may reflect a lower proliferative profile.

## Introduction

Acromegaly is a rare disease caused by a persistent excess of growth hormone (GH) and its main mediator, insulin-like growth factor-1 (IGF-1). Most cases are secondary to a GH-secreting pituitary adenoma, which is a macroadenoma in almost 70% of cases (1, 2). Acromegaly is associated with increased morbidity and mortality and reduced quality of life; therefore, its prompt diagnosis and proper management are mandatory (3–5). Historically, guidelines recommend measuring serum IGF-1 in the suspicion of disease and, in patients with elevated IGF-1 levels, confirming the diagnosis by demonstrating an unsuppressed GH value following documented hyperglycemia during an oral glucose tolerance test (OGTT) (1). GH values indicative of insufficient suppression have progressively lowered over the last decades with the improvements and availability of ultrasensitive assays for GH measurement, up to 0.4 μg/L (1, 6, 7).

Nevertheless, several reports documented the presence of clinically evident acromegalic patients with apparently normal GH secretion (8–16), a condition first defined as “micromegaly” by Dimaraki et al. (10), also called “low GH-acromegaly” (17). The definition of normal GH secretion differs between studies, and little is known about the pathogenesis and clinical progression of this condition. In a previous study (9), we retrospectively characterized a group of patients presenting with high IGF-1 levels but suppressed GH nadir < 0.4 μg/L; among them, we identified a subgroup characterized by suggestive acromegalic features (“micromegalics”), leading to speculations on their clinical management. Moreover, these patients often display a peculiar demographical and clinical presentation (9, 13, 17), especially with respect to their radiological features; notably, a pituitary adenoma is not always identified, increasing difficulty in their management.

Furthermore, the most recent consensus on disease management (18) proposes to confirm the diagnosis of acromegaly in patients with typical clinical signs and symptoms and IGF-1 > 1.3, the upper limit of normal (ULN), with no need to measure GH levels after glucose load, underlying how there is no cutoff for glucose-suppressed GH that definitively excludes the diagnosis, further supporting our observations but also posing the challenge of how to classify and manage micromegaly.

In the present study, we applied a systematic screening program to detect comorbidities in micromegalic patients and compared the results to a matched group of acromegalic patients with elevated GH levels. Moreover, we first collected preliminary data on the treatment response of a small subgroup of micromegalic patients and performed laboratory analysis on their tissue samples, aiming to better characterize this rare entity.

Despite the lack of uniformity in the definition of “micromegaly,” mostly reflecting assay availabilities and study periods, in the present work, we will use this terminology to refer to patients with IGF-1 levels above the age- and sex-adjusted ULN and GH nadir < 0.4 µg/L using ultrasensitive assays (1, 19), associated with typical clinical features of acromegaly, as defined by previous authors (10, 11, 14).

## Materials and methods

### Population

We enrolled 30 adult patients with micromegaly (including 25 patients selected from our previously published cohort based on a clinically suggestive acromegalic phenotype (9); median age: 59 years, IQR: 53.5–65, 73.3% females) and 30 matched patients with a diagnosis of acromegaly (median age 60 years, IQR 51–66, 73.3% females), referred to a single tertiary endocrinology unit (Fondazione IRCCS Ca’ Granda Ospedale Maggiore Policlinico, Milan) between 2006 and 2022. Patients with micromegaly presented with IGF-1>1ULN and GH nadir <0.4 µg/L after OGTT, while patients with acromegaly displayed inadequate GH suppression (>0.4 µg/L) using ultrasensitive assays (1, 18, 19). Patients with acromegaly were matched for age and sex to be able to compare the prevalence and burden of acromegaly-related comorbidities between the two groups.

Exclusion criteria were represented by all conditions that may alter the GH-IGF-1 axis, such as pregnancy, hepatic and renal failure, chronic inflammation, malnutrition, Cushing’s disease, dopamine-agonist use, and undergoing estrogen therapy (7, 20–22).

In both cohorts, IGF-1 levels were evaluated because of a clinical suspicion of acromegaly, with patients presenting suggesting features. The presence of typical acromegalic features was defined in the presence of at least two physical changes (including the prominence of the brow, prognathism, macroglossia, and nose and lip enlargement), determined by board-certified experts in endocrinology with at least 5 years of clinical practice.

### Methods

We retrospectively analyzed hormonal data collected at the time of diagnosis, including IGF-1, GH nadir (GHn), defined as the lowest GH value at any time during a 2h OGTT [blood samples for GH were collected at 30, 60, 90, and 120 min after a standard 75 g glucose load, and GH random levels (GHr) were assessed fasting in the morning before starting OGTT]. IGF-1 values were compared with appropriate age- and sex-adjusted ranges. IGF-1 is expressed as standard deviation scores (SDS) and a percentage of the ULN for individual laboratory samples. High levels of IGF-1 were confirmed in at least two blood samples.

Following our previous findings (9), all micromegalic patients underwent a systematic screening program to detect acromegaly related comorbidities within 24 months from the first clinical assessment, including arterial blood pressure evaluation, glucose metabolism assessment (fasting blood glucose, 120 min post-OGTT blood glucose, and glycated hemoglobin), thyroid ultrasound for goiter, colonoscopy for colonic polyposis, echocardiography for cardiopathy, and the Epworth Sleepiness Scale (ESS) questionnaire for obstructive sleep apnea syndrome (OSAS) followed by polysomnography if ESS ≥ 10. According to the international criteria, we considered as glucose metabolism alteration the presence of impaired fasting glucose (IFG), impaired glucose tolerance (IGT), and diabetes mellitus (DM) (1, 23). The screening of comorbidities was already available in patients with acromegaly, as indicated by good clinical practice, and data included in this study were collected following the diagnosis of acromegaly.

Finally, we included the results of magnetic resonance imaging (MRI) of the sellar region before and after gadolinium contrast, registering the presence of a pituitary adenoma, defined as macro (≥10 mm) or microadenoma (<10 mm).

### Assays

Serum GH was assayed at Fondazione IRCCS Ca’ Granda Ospedale Maggiore Policlinico, Milan, by a chemiluminescence method (Immulite 2000, Siemens Medical Solutions Diagnostics, detection limit of 0.01 μg/L). Standards used for calibration were IS 80/505 from 2006 to July 2010 and IS 98/574 from August 2010. IGF-1 was measured by a chemiluminescent immunometric assay (Immulite 2000 IGF-1; Siemens Medical Solutions Diagnostics), and standards used for calibration were IRR 87/518 from 2008 to April 2017 and IS 02/254 from May 2017.

### Follow-up and treatment details

Treatment was proposed to micromegalic patients with IGF-1 levels > 1.2 ULN with a growing trend during follow-up and at least three typical comorbidities (**Table 1**). Two patients showing a pituitary adenoma at MRI scan underwent transsphenoidal surgery (TSS), which was performed at the Neurosurgery Unit of Fondazione IRCCS Ca’ Granda Ospedale Maggiore Policlinico, Milan. Two patients with normal pituitary MRI scans were started on medical treatment with somatostatin analogues (SSAs).

**Table 1:** Acromegaly related comorbidities at baseline in the two groups.

	Micromegaly	Acromegaly	*P*-value
Comorbidities per patient, median *n* (IQR)	4 (3–5)	5 (4–5)	0.43
Goiter, *n* (%)	22/26 (84.6%)	23/29 (79.3%)	0.73
Carpal tunnel, *n* (%)	12/30 (40%)	12/30 (40%)	1.00
Colonic polyps, *n* (%)	11/17 (64.7%)	17/24 (70.8%)	0.19
Malignancies, *n* (%)	10/30 (33.3%)	11/30 (36.7%)	1.00
Hypertension, *n* (%)	18/30 (60%)	22/30 (73.3%)	0.41
Arrhythmia, *n* (%)	6/30 (20%)	8/30 (26.7%)	0.76
Cardiopathy, *n* (%)	19/24 (63.3%)	22/28 (73.3%)	0.57
- Ventricular hypertrophy, *n* (%)	11/24 (45.8%)	18/28 (64.3%)	0.26
- Valve disease, *n* (%)	4/24 (16.7%)	12/28 (42.9%)	**0.006**
- Diastolic dysfunction, *n* (%)	5/24 (20.8%)	12/28 (42.9%)	0.13
Ischemic heart disease, *n* (%)	5/30 (16.7%)	7/30 (23.3%)	0.74
Obstructive sleep apnea syndrome, *n* (%)	7/30 (23.3%)	4/30 (13.3%)	0.5
Glucose alterations, *n* (%)	20/30 (66.7%)	25/30 (83.3%)	0.23
Diabetes mellitus, *n* (%)	7/30 (23.3%)	17/30 (56.7%)	**0.016**

Data are expressed as number (*n*) and percentages (%) or inter-quartile ranges (IQR).

Comparison analyses were assessed considering only the patients screened for each comorbidity, as detailed (i.e., had a thyroid ultrasound or colonoscopy performed).

Bold values indicate statistically significant results (p < 0.05)

The first biochemical evaluation after treatment was performed 3 months after surgery in the first two patients and on the day of the fourth administration of SSA in the remaining two cases. Clinical and biochemical evaluations were performed at every subsequent follow-up visit every 6 months. Clinical symptoms and quality of life were assessed by the following self-administered questionnaires: 36-Item-Short-Form Health Survey (SF-36), measuring physical and psychological well-being (24), and Acromegaly QoL Questionnaire (AcroQoL) (25).

Post-surgery MRI to assess radiological outcomes was performed 3 months after surgery and 12 months thereafter.

### Western blot analysis

Total proteins extracted from surgically removed pituitary tumors were quantified by BCA assay, separated on SDS/polyacrylamide gels, and transferred to a nitrocellulose filter. The GH antibody (Boster, Pleasanton, CA) was used at 1:1000, and the Cyclin D3 antibody (Cell Signaling, Danvers, MA) was used at 1:2000. The membrane was stripped and reprobed with an anti-GAPDH antibody (Ambion, Thermo Fisher Scientific, Waltham, MA) as a housekeeping gene at 1:4000 for 1h at room temperature. Secondary antibodies, anti-mouse or anti-rabbit (Cell Signaling Technology, Danvers, MA), were incubated at room temperature for 1h at 1:2000. Chemiluminescence was detected using the ChemiDOC-IT Imaging System (UVP, Upland, CA) and analyzed with NIH ImageJ software.

### Measurement of GH levels

Human pituitary cells were obtained by TSS surgery from two micromegalic and three acromegalic patients. Tissues were enzymatically dissociated in DMEM (Sigma-Aldrich, St. Louis, MO) containing 2 mg/ml collagenase (Sigma-Aldrich, St. Louis, MO) at 37°C for 2h, as previously described (26). Dispersed cells were cultured in DMEM supplemented with 10% FBS, 2 mM glutamine, and antibiotics (Gibco, Invitrogen, Life Technologies Inc., Carlsbad). For GH secretion quantification, cells were incubated in complete medium for 4h at 37 °C and 5% CO_2_. GH in culture media was measured by a specific chemiluminescent immunometric assay (Immulite 2000, Siemens Medical Solutions Diagnostics, Los Angeles).

### Statistical analysis

We described continuous parameters with normal distribution as mean ± standard deviation (SD) and non-Gaussian distributions as median with interquartile range (IQR). Continuous and non-Gaussian data were compared using a *t*-test and Wilcoxon–Mann–Whitney test, respectively. Categorical data were presented as percentage (%) and proportion (/) and analyzed using the Chi-squared test or Fisher’s exact test as appropriate. *P*-values < 0.05 were considered statistically significant. Statistical analyses were conducted using GraphPad Prism 7.0 (GraphPad Software) and IBM SPSS 28.0 (IBM Analytics).

## Results

### Clinical and hormonal features of the two groups

The main features of the two groups at the time of diagnosis are detailed in **Table 2**. As mentioned above, all micromegalic patients showed high IGF-1 levels (median IGF-1 ULN: 1.2, IQR 1.1–1.4). Acromegalic patients showed significantly higher median levels of IGF-1 (436.5 vs. 250 μg/L, *p* < 0.01), as well as of median GHn and GHr values, as expected (6 vs. 0.15 and 8.6 vs. 2.7 μg/L, respectively, for both *p* < 0.01).

**Table 2:** Clinical and hormonal data of the two groups at the time of diagnosis.

	Micromegaly	Acromegaly	*P*-value
Demographic			
Age, years	59 (53.5–65)	60 (51–66)	0.96
Sex (M/F)	8/22	8/22	NS
Pituitary MRI			
Macroadenoma, *n* (%)	2 (8.6)	21 (70)	**< 0.001**
Microadenoma, *n* (%)	8 (34.8)	9 (30)	0.77
Normal, *n* (%)	5 (21.8)	0 (0)	**0.01**
Other, *n* (%)	8 (34.8) *	0 (0)	**< 0.001**
Hormonal values			
IGF-1 values, μg/L	250 (226–287.5)	436.5 (349.8–796.8)	**< 0.01**
IGF-1, +SDS	3.2 (2.6–4.2)	8.3 (6.5–18.8)	**< 0.01**
IGF-1, ULN	1.2 (1.1–1.4)	2.2 (1.9–3.7)	**< 0.01**
GHn, μg/L	0.15 (0.09–0.3)	6 (2.3–14.6)	**< 0.01**
GHr, μg/L	2.7 (0.7–5.5)	8.6 (3.9–21.5)	**< 0.01**

Data are expressed as median (interquartile ranges - IQR).

SDS, standard deviation scores; ULN, upper limit of normal; GHr, GH random; GHn, GH nadir. MRI was available in 23/30 micromegalic patients and 30/30 acromegalic patients.

*Two patients with empty sella, one pituitary hyperplasia, and five pituitary stalk deviation with no signs of adenoma.

Bold values indicate statistically significant results (p < 0.05).

Focusing on neuroradiological findings, all acromegalic patients presented a pituitary adenoma, which was a macroadenoma in 21/30 (70%) patients. On the contrary, most micromegalic patients (52%) did not show any pituitary adenoma at MRI scan, where we registered only two macroadenomas, eight microadenomas, and one pituitary hyperplasia (see **Table 2**).

Considering the newly proposed diagnostic criteria of acromegaly to confirm the diagnosis without needing to perform OGTT in cases of IGF-1 > 1.3 ULN (18), all acromegalic patients of our group meet these criteria. On the contrary, only 10/30 (33%) in the micromegalic group showed IGF-1 > 1.3 ULN at diagnosis, with only 50% of them displaying a pituitary lesion (four microadenomas and one hyperplasia) at MRI. Focusing on GH nadir values, applying sex- and BMI-adapted GHn cutoffs (18, 27), suggesting the use of a lowered threshold of 0.2 μg/L in overweight patients, 7/30 (23.3%) patients in the micromegalic group would be reclassified as acromegalic.

### Acromegaly related comorbidities in the two groups: comparative analysis

The prevalence of comorbidities in the two groups of patients is detailed in **Table 1**. Interestingly, no differences were found in the prevalence of specific acromegalic complications between acromegalic patients and micromegalic ones, except for valve disease and diabetes mellitus, which resulted as significantly more prevalent in the acromegalic cohort (*p* = 0.006 and *p* = 0.016, respectively).

### Follow-up and treatment outcomes in micromegaly

The longitudinal assessment confirmed the data of our previous study (9). In untreated micromegalic patients, we confirmed the overall stability of the hormonal profile, with no patient meeting the diagnostic criteria of acromegaly after a mean follow-up time of 6.2 ± 3.7 years. Pituitary MRI results remained stable as well, except for one patient showing radiological progression from micro to macroadenoma (Patient N.1 in **Table 3**, see below).

**Table 3:** Clinical data of the four patients selected for treatment, treatment details and follow-up.

*N*, sex	Age (years)	Before treatment				Comorbidities	Type of therapy	After treatment			Follow-up (years)
		Adenoma	IGF-1 µg/L (SDS)	GHr (µg/L)	GHn (µg/L)			IGF-1 µg/L (SDS)	GHr (µg/L)	GHn (µg/L)	
1, F	58	Yes (macro)	250 (+3.2)	8.8	0.26	MNG, colorectal adenomas and dolichocolon, recurrent rectal NET, mitral and tricuspid valve insufficiency, arrhythmia, IGT	TSS	139 (+0.6)	0.25	0.08	2
2, F	59	Yes (micro)	220 (+2.5)	3.4	0.09	MNG, colonic adenocarcinoma, OSAS, AH, IFG, IGT	TSS	124 (+0.1)	< 0.05	< 0.05	4
3, M	56	No	328 (+5.4)	0.48	0.31	MNG, colorectal adenomas, small bowel NETs, AH, ICM, OSAS, IFG, IGT	LAN	154 (+0.8)	2	NA	3
4, F	58	No	342 (+5.3)	0.89	0.17	GMN, BCC, Pheochromocytoma, AH, cardiac diastolic dysfunction, ICM, PHPT, IGT	OCT	185 (+1.6)	1.7	NA	7

*N*, number; F, female; M, male; GHr, GH random; GHn, GH nadir; MNG, multinodular goiter; NET, neuroendocrine tumor; IGT, impaired glucose tolerance; IFG, impaired fasting glucose; OSAS, obstructive sleep apnea syndrome; AH, arterial hypertension; ICM, ischemic cardiopathy; BCC, basal cell carcinoma; PHPT, primary hyperparathyroidism; TSS, transsphenoidal surgery; LAN, lanreotide autogel; OCT, octreotide LAR.

We proposed treatment to patients with IGF-1 > 1.2 ULN presenting at least three comorbidities. Among patients with these characteristics, one died during follow-up, and two refused treatment. Four patients were therefore treated, with their clinical picture detailed in **Table 3**. Patients N.1 and N.2 presented a well-defined pituitary adenoma at MRI scan (one micro- and one macroadenoma) and hence underwent TSS. At pathology evaluation, they both resulted in a histologically proven somatotroph adenoma (GH+ at immunohistochemical stain), and no residual pathological tissue at post-surgery scan was detected. Tissue samples from the two tumors were also collected and further analyzed. Patients N.3 and N.4 conversely, presented neuroimaging negative for pituitary adenoma yet highly increased IGF-1 (1.6 ULN and 2.1 ULN, respectively) and were therefore started medical treatment with SSAs (Lanreotide autogel 90 mg every 28 days and Octreotide LAR 30 mg every 28 days, respectively). All patients demonstrated normalization in IGF-1 after treatment. Patients N.1 and N.2 also showed a reduction in GH levels after surgery (GHn < 0.1). At follow-up, all patients reported subjective improvement in physical and psychological well-being, as objected to through self-administered questionnaires.

### GH isoforms analysis and proliferative rate evaluation

**Figure 1:**
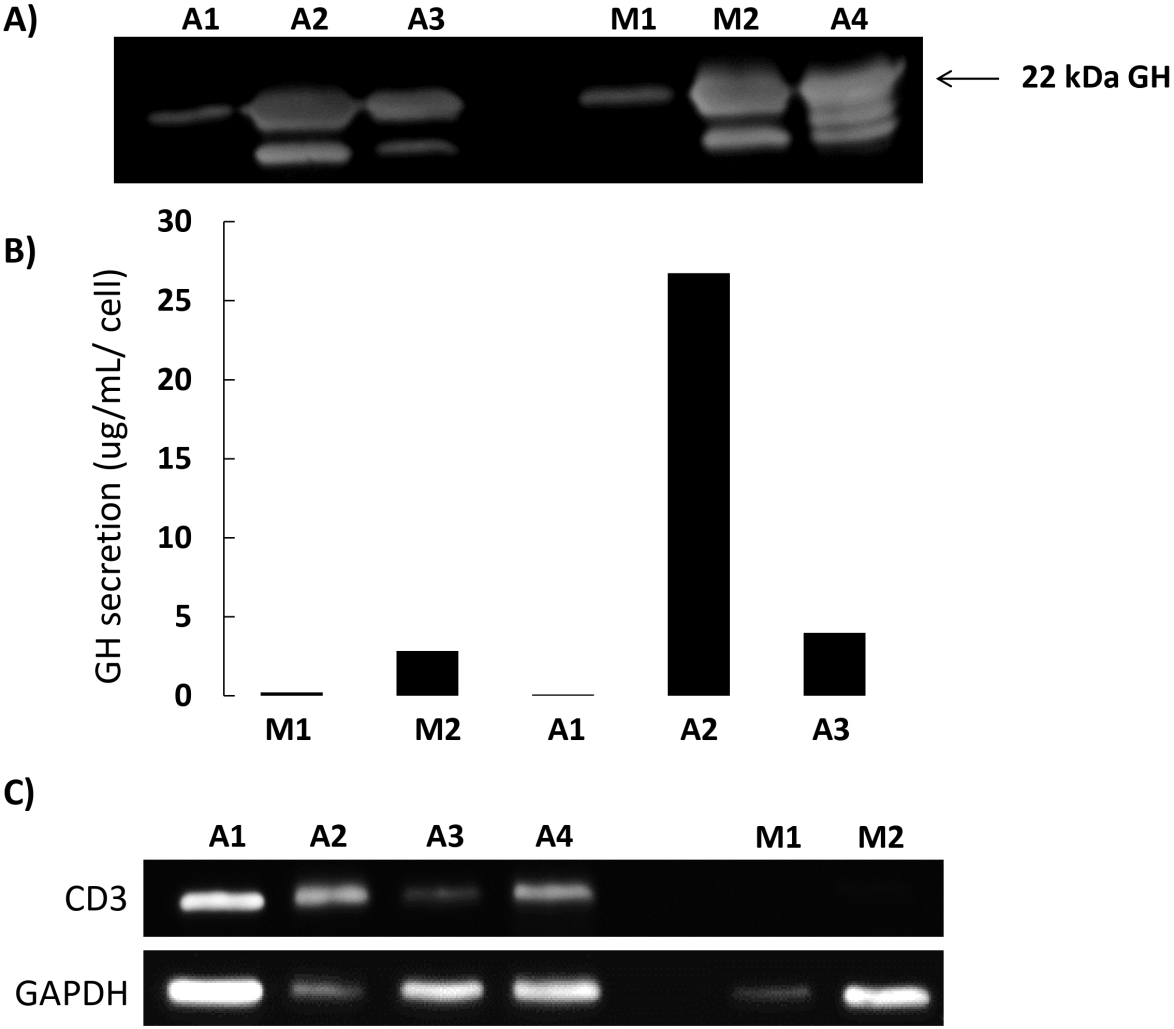
Laboratory analysis on tissue samples. **(A)** Immunoblot of GH in GH-secreting tumors. Total proteins were extracted from tumor tissues. Membrane was incubated with GH antibody. **(B)** GH secretion: the graph shows the GH levels in the culture medium of primary cultured pituitary tumoral cells, normalized on the number of cells. Cells were incubated in complete medium for 4h. **(C)** Immunoblot of cyclin D3 (CD3). Membrane was incubated with CD3 antibody. GAPDH was used as a loading control.

To evaluate the expression of different isoforms of GH, we analyzed total proteins extracted from two micromegalic patients’ tumor tissues (M1, M2) in comparison with four tumors from known acromegalic patients (A1-4) by Western blot analysis. As shown in **Figure 1A**, the major pituitary GH isoform of 22 KDa (22-k) was expressed in all samples analyzed. In addition, a lower molecular weight isoform was detectable in M2 and in three out of four acromegalic patients, probably consistent with the 20 KDa GH that derives from alternative splicing and represents the second most abundant GH isoform. An additional GH isoform was present in the A4 sample.

We then quantified the amount of GH secreted in cell culture medium (**Figure 1B**). The isoform specificity of this assay has not been characterized. Our results showed a variable secretion of GH from the different tumors, with results that were comparable to those obtained by Western blot analysis.

In order to indirectly evaluate the proliferative rate of these tumors, we tested the expression of cyclin D3, a major regulator of cell cycle progression (**Figure 1C**). Our data showed that cyclin D3 was expressed in all acromegalic patients’ tumors but was nearly undetectable in M1 and M2.

## Discussion

In a previous study, we described a large cohort of patients presenting with high IGF-1 and suppressed GHn <0.4 μg/L, and among them we identified two different subgroups according to the presence (micromegaly) or absence of acromegalic features, demonstrating different clinical implications in the two cohorts. Whilst patients with high IGF-1 screened for other pituitary disorders in the absence of clinical suspicion likely reflect a dosage interference or inadequate cut-off of IGF-1 with no relevant clinical significance (9, 15), patients with micromegaly presented a higher rate of symptoms and comorbidities with a clinical worsening over time. Therefore, we decided to systematically assess acromegaly related comorbidities in these patients.

In the present study, we compared clinical data of patients with micromegaly with a cohort matched for age and sex of acromegalic patients, finding a comparable rate of comorbidities in the two groups. These results confirm the importance of maintaining a close screening follow-up. Moreover, despite the similar prevalence of glucose alteration, micromegalic patients displayed a significantly lower rate of diabetes, leading to speculation on a milder clinical impact of disease, possibly linked to the low GH secretion. It is indeed well known how GH and IGF-I exert different effects on glucose metabolism, with GH being the main leading actor in the pathogenesis of diabetes (28).

Despite the high rate of hypertension and cardiopathies, the prevalence of cardiac valve disease resulted significantly lower in micromegaly. Valve disease has been reported in 20%–80% of patients with acromegaly (29, 30), and it has mainly been referred to as direct GH effects on regulation of the extracellular matrix, including expression of metalloproteinases, proteoglycan synthesis, and the deposition of collagen and mucopolysaccharides (31); hence, we could still speculate about a milder impact in micromegalic patients with low GH secretion.

Moreover, in accordance with previous reports (11, 13), micromegaly is typically characterized by an older age at diagnosis and distinct radiological findings. Since our cohorts were matched for age, no comparison regarding age at diagnosis can be drawn from the present analysis. However, the neuroradiological differences observed in our series were consistent with previous reports, being characterized by smaller adenomas or even normal pituitary imaging in the majority of patients. Finally, at longitudinal assessment, patients did not show progression toward the biochemical profile of classical GH-hypersecretory acromegaly, arguing against the hypothesis that micromegaly simply represents an early stage of the disease.

The most recent consensus on acromegaly proposes to confirm the diagnosis in patients with a typical clinical picture and IGF-I>1.3 ULN, with no need to measure GH levels after OGTT (18). Applying these proposed criteria to our cohort, 33% of patients with micromegaly would be classified as acromegalic. In addition, using the proposed GHn cutoffs from the Consensus (0.2 μg/L in overweight patients (18)), 23% of our micromegalic cohort would also be considered acromegalic. However, there is still a large percentage of patients with acromegalic features and lower GH nadir values who do not meet the new proposed diagnostic criteria but still require careful screening and follow-up. Furthermore, the differences in epidemiological, clinical, and neuroradiological findings make their management challenging. Notably, among the 10 patients with IGF-1 > 1.3 ULN (18), 50% displayed a negative neuroradiological image. These findings highlight how the main challenge in micromegaly is not only disease classification but also the definition of appropriate treatment strategies for patients who remain outside conventional diagnostic frameworks.

The pathophysiology of micromegaly remains unclear to date. Since our previous work, a few new studies addressing this issue have been published, suggesting, for example, a possible role of advanced glycation end products in stimulating IGF-1 secretion independently of GH levels (32), yet this remains to be proven. Other proposed mechanisms include an enhanced peripheral sensitivity to GH in patients with specific GH-receptor isoforms (33), an increase in IGF-1 production caused by continuous exposure of the liver to minimally elevated tonic GH levels (8, 10), or the secretion of other active GH isoforms not detected by common GH assays, which mainly measure the 22-k circulating polypeptide.

To our knowledge, this is the first study reporting tissue-level analyses in patients with a micromegalic phenotype. Although limited by the small number of available samples, these data provide a first opportunity to explore some of the pathogenetic hypotheses proposed. The hypothesis of biologically active GH isoforms not adequately detected by conventional assays is particularly intriguing, but most commercially available GH assays and antibodies have not been fully characterized for their cross-reactivity with the various GH isoforms. In our study, the antibody used for GH isoform analysis recognized both the 22-kDa and 20-kDa isoforms. We found expression of the 22-kDa isoform in all tumor samples, whereas the 20-kDa isoform was absent in one micromegalic and one acromegalic sample; an additional isoform was detected in a single acromegalic tumor. Although these findings do not support major differences in the detectable GH isoform profile between micromegalic and acromegalic tumors, they do not allow us to exclude the presence of other biologically active GH isoforms not recognized by the antibody employed.

GH secretion patterns resulted quite variable in both acromegalic and micromegalic patients. Interestingly, our results showed that micromegaly differed from acromegaly in the expression of cyclin D3, a major regulator of cell cycle progression in pituitary cells. Indeed, cyclin D3 showed a variable expression in acromegalic tissues, but it was nearly undetectable in micromegalic ones, suggesting a low proliferative rate of tumoral cells in these patients. This observation could possibly account for the older age at diagnosis registered in micromegaly (11, 13), as well as its smaller adenomas observed at neuroimaging. However, the limited number of available samples precludes definitive conclusions and requires confirmation in larger studies.

We proposed treatment to patients with IGF-1 > 1.2 ULN, as these levels were demonstrated to correlate with poor outcomes in treated patients (34), with an increasing trend and comorbidities. Our positive results in a small cohort demonstrated that micromegalic patients can benefit from either medical or surgical therapy, supporting the idea of treating them as classic acromegalic patients. However, our data are still preliminary and limited to a very small subgroup of patients, which represents the main limitation of this study. Nonetheless, the rarity of the condition and the difficulties related to proposing off-label treatment in the absence of a clear diagnosis according to diagnostic guidelines support the importance of collecting even preliminary data to help better understand this entity.

In conclusion, while acromegaly is currently a well-addressed disorder with multiple treatment options, micromegaly remains poorly understood. Since it displays similar rates of comorbidities as acromegaly, a close clinical follow-up is recommended. Treatment should be considered in selected patients, as it appears to improve overall clinical well-being parameters, with individualized approaches based on each patient’s characteristics.

## Institutional review board statement

The study was conducted in accordance with the Declaration of Helsinki. The local ethics committee (Milan, approval number 973_2019bis) approved this protocol study.

## Data Availability

The original laboratory data, including the original images underlying the reported experiments, have been deposited in the University of Milan Dataverse repository and are publicly available at https://doi.org/10.13130/RD_UNIMI/JHVBWK. The clinical datasets will be made available by the corresponding author upon reasonable request.
